# Clinical Profile and Outcomes of Pulmonary Embolism in Central Iran: A Retrospective Cohort Study

**DOI:** 10.34172/aim.31907

**Published:** 2024-12-01

**Authors:** Abbas Andishmand, Leila Sharifi, Seyedeh Mahdieh Namayandeh

**Affiliations:** ^1^Yazd Cardiovascular Research Center, Shahid Sadoughi University of Medical Sciences, Yazd, Iran

**Keywords:** Mortality, Pulmonary embolism, Thrombolytic therapy, Venous thromboembolism

## Abstract

**Background:**

Pulmonary embolism (PE) is a significant public health concern. This retrospective cohort study examines the clinical profiles and outcomes of patients diagnosed with PE at a medical center in central Iran, aiming to identify mortality predictors during hospitalization and follow-up.

**Methods:**

Data from 109 patients diagnosed with PE were analyzed, with a median follow-up of 23 months. The collected information included demographic and clinical characteristics, laboratory findings, treatment protocols, and outcomes. Logistic regression and Kaplan-Meier survival analysis were used to identify independent mortality predictors and assess survival impact.

**Results:**

The mean age was 59.2 years (±19.7), with 51.4% male. Common symptoms included dyspnea (86%) and chest pain (53%), with non-massive PE being the most prevalent (63%). Independent mortality predictors identified were age (odds ratio [OR] 1.065 per year, *P*<0.001), female sex (OR 4.421, *P*=0.009), and PE severity (OR 0.262, *P*=0.023). Kaplan-Meier analysis showed reduced survival probabilities in females (*P*=0.009), those with provoked PE (*P*=0.002), patients over 65 (*P*=0.016), and individuals with comorbidities (*P*=0.018). In-hospital mortality was 10.1%, linked to provoked massive PE, absence of thrombolytic therapy, and reduced left ventricular ejection fraction (LVEF).

**Conclusion:**

In this cohort, age, sex, and PE severity were significant mortality predictors, while provoked PE, advanced age, and comorbidities were associated with lower mid-term survival probabilities.

## Introduction

 Pulmonary embolism (PE), a serious medical condition arising from thrombotic obstruction of the pulmonary vasculature.^1^ It continues to present a significant challenge to healthcare systems despite advances in diagnosis and treatment.^2^ Anticoagulant therapy remains the cornerstone of PE management, yet optimal treatment strategies are still debated.^3^ Furthermore, recent data from the United States reveal an alarming trend of increasing hospitalization rates and mortality attributed to PE, particularly among older adults.^4,5^ This underscores the critical need for further research to deepen our understanding of PE pathophysiology and to develop more effective and personalized therapeutic approaches.^6^

 This study had two primary objectives. First, to provide a comprehensive characterization of the demographic profile, risk factors, and clinical presentation of patients hospitalized for PE. Second, to evaluate the short- to mid-term outcomes in this patient population. By achieving these objectives, this study aimed to contribute to the existing body of knowledge regarding PE, ultimately seeking to enhance risk stratification, optimize treatment strategies, and improve patient outcomes and prognosis.

## Materials and Methods

 This retrospective cohort study investigated the risk factors for mortality and treatment outcomes in patients with PE. Data were collected from the electronic medical records (EMRs) of patients admitted with a primary diagnosis of PE to Afshar Hospital, a tertiary care center in Yazd, Iran, between 2018 and 2020.

###  Study Population

 The study population consisted of adult patients ( ≥ 18 years) admitted to the hospital with a primary diagnosis of PE during the specified study period. Inclusion criteria were broad, encompassing patients with concurrent deep vein thrombosis (DVT) and those with a history of PE. Patients who died before the initiation of any treatment were excluded from the analysis.

 PE diagnosis was established through a multi-faceted approach incorporating clinical evaluation and diagnostic testing. This process included laboratory analyses, echocardiography, pulmonary angiography, and radionuclide lung perfusion scans. In cases presenting with severe hemodynamic compromise or imminent cardiac arrest, the diagnosis was primarily established based on clinical presentation and laboratory findings, often with the adjunct of echocardiography. This expedited approach was necessitated by the critical need for immediate therapeutic intervention and the infeasibility of performing more invasive diagnostic procedures in such unstable patients.

 All patients received intravenous heparin as a component of their anticoagulation therapy during their hospitalization. Before discharge, the attending physician prescribed individualized anticoagulation regimens, which included warfarin or non-vitamin K antagonists, tailored to each patient’s specific requirements.

###  Sample Size 

 The sample size for this prospective cohort study comprised 109 patients. This number was determined based on a power analysis conducted to ensure sufficient statistical power for detecting clinically significant differences in the primary outcomes related to PE, considering the specific research objectives and the inherent limitations of a single-center study design. While acknowledging the constraints imposed by the study setting, the power analysis supports the validity of the findings obtained.

###  Data Acquisition and Management

 The data utilized in this study were derived from electronic medical records (EMRs) and hospital databases. Variables of interest included patient demographics, medical history, clinical presentation, laboratory and pulmonary computed tomography angiography (CTA) findings, treatment interventions, and short- to mid-term outcomes. To ensure accuracy and consistency, trained research personnel were responsible for data collection and management.

###  Data Analysis

 Statistical analyses were conducted utilizing SPSS version 22 (IBM Corp., Armonk, NY, USA). Continuous variables were expressed as means ± standard deviations, while categorical variables were presented as counts and percentages. One-way analysis of variance (ANOVA) was employed to compare continuous variables between groups. Chi-square or Fisher’s exact tests were utilized, as appropriate, for the analysis of categorical variables. Logistic regression analysis was performed to identify predictors of the primary outcomes. Survival distributions were estimated using Kaplan-Meier methods and compared between groups via the log-rank test. Statistical significance was defined as a *P* value < 0.05. The primary outcomes of interest were in-hospital mortality, recurrence of pulmonary thromboembolism, and bleeding events.

## Results

###  Patient Demographics and Baseline Clinical Presentation

 A total of 109 patients diagnosed with PE were included in the study. The study population had a mean age of 59.2 years ( ± 19.7 years), with a balanced gender distribution (51.4% male, N = 56). Upon presentation, the average arterial blood pressure was 110/70 mm Hg, and the mean heart rate was 100 beats per minute. The most frequently reported symptom was dyspnea, present in 86% of the patients (N = 94), followed by chest pain, which was reported by 53% (N = 58).

###  Diagnostic Approaches and Clinical Characteristics 

 In a study investigating PE, computed tomographic pulmonary angiography (CTPA) emerged as the predominant diagnostic modality, employed in 73.3% of the patient cohort (N = 80). Alternative diagnostic strategies, including clinical evaluation and ventilation-perfusion scans, were utilized in 23.9% (N = 26) and 2.8% (N = 3) of patients, respectively. Non-massive PE (a combination of small and sub-massive) represented the most prevalent form of the condition, comprising 63.7% (N = 69) of the diagnosed cases.

 Etiological classification revealed that 61.5% (N = 67) of the participants presented with provoked embolism, characterized by the presence of identifiable risk factors for thromboembolic events. Conversely, 38.5% (N = 42) of the individuals were classified as having unprovoked pulmonary thromboembolism, indicating the absence of discernible triggering factors.

 Electrocardiographic (ECG) abnormalities were a frequent finding in this patient population, observed in 85.3% (N = 93) of the cases. The classic S1Q3T3 pattern, indicative of right ventricular strain, was documented in 16% (N = 18) of the ECGs. Sinus tachycardia, a common manifestation of PE, was present in 44% (N = 48) of the patients. Notably, the most prevalent ECG abnormality was the presence of pathological Q waves in lead III, detected in 55% (N = 60) of the individuals.

 Laboratory analysis revealed an average D-dimer level of 6648 ng/mL ( ± 2476) and a mean troponin level of 8431 ng/L ( ± 1512). Echocardiography demonstrated normal ejection fraction in the majority of patients (82.6%, N = 89). The hemodynamic assessment indicated a mean systolic pulmonary artery pressure of 41 mm Hg, with a maximum value of 95 mm Hg. Recombinant tissue plasminogen activator (r-PA) therapy was administered to 18.3% (N = 20) of the total cohort. Further details are available in Table 1.

**Table 1:** Characteristics and Clinical Profile of Patients with Pulmonary Embolism

**Variable**	**Value**
Age (years, mean ± SD)	59.2 ± 19.7
Sex- n (%)	
Male	56 (51.4%)
Female	53 (48.6%)
Risk factor, n (%)	
Malignancy	5 (10.2%)
Pregnancy	2 (4.1%)
CHF	4 (8.2%)
CKD	2 (4.1%)
Prior VTE	8 (16.3%)
Recent surgery	10 (20.4%)
OCP consumption	4 (8.2%)
Prolonged Bed rest	10 (2.4%)
Lower Limb Immobility	15 (30.6%)
Recent infection	1 (0.9%)
PE severity- n (%)	
Non-massive	69 (63.3%)
Massive	40 (37.7%)
Treatment strategy- n (%)	
Anticoagulant therapy	80 (73.3%)
Thrombolytic therapy	29 (27.7%)
Symptoms/presentation- n (%)	
Cardiac arrest (Out of hospital)	3 (2.7%)
Dyspnea	94 (86.2%)
Chest pain	58 (53.2%)
Syncope	25 (23%)
Lightheadedness	12 (11.0%)
Palpitation	10 (9.2%)
Hemoptysis	5 (4.5%)
Hemodynamic parameter (mean ± SD)	
Systolic blood pressure (mm Hg)	114.4 ± 21.3
Diastolic blood pressure (mm Hg)	74.7 ± 14.0
Heart rate (beats/min)	98.5 ± 17.8
O_2_ sat (%)	90.7 ± 4.8
Lab test (mean ± SD)	
Hb (g/dL)	13.2 ± 2.3
WBC (10^3^/µL)	10.3 ± 3.6
Platelet count (10^3^/µL)	229.7 ± 97.6
BUN (mg/dL)	39.7 ± 17.9
Cr (mg/dL)	1.3 ± 0.49
Troponin (ng/L)	8431 ± 1512
D-dimer (ng/mL)	6649 ± 2477
Diagnostic modality- n (%)	
Pulmonary CTA	80 (73.3%)
Clinical	26 (23.9%)
Perfusion scan	3 (2.8%)
PE category- n (%)	
Provoked	67 (61%)
Unprovoked	42 (39%)
ECG changes- n (%)	
Sinus tachycardia	48 (44%)
Q in lead III	60 (55%)
S in lead I	32 (29%)
Inverted T wave (lead III)	56 (51%)
Inverted T wave (precordial leads)	37 (34%)
RBBB	21 (17%)
S in lead V5, V6	14 (13%)
Echocardiogram parameter (mean ± SD)	
LVEF (%)	51.1 ± 6.3
PASP (mm Hg)	41.2 ± 19.5

CHF: Congestive heart failure, CKD: Chronic kidney disease, VTE: Venous thromboembolism, PE: Pulmonary embolism, CTA: Computed tomography angiogram, ECG: Electrocardiogram, RBBB: Right bundle branch block, OCP: Oral contraceptive pill, LVEF: Left ventricular ejection fraction, PASP: Pulmonary arterial systolic pressure, WBC: White blood cell count, Hb: Hemoglobin, HR: Heart rate, BUN: Blood urea nitrogen, Cr: Creatinine, SD: Standard deviation.

###  Follow-up Duration and Clinical Outcomes 

 Patients were followed for a mean duration of 22.1 months (SD = 13.1), with a median follow-up duration of 23.0 months (range: 0-45 months). Clinical outcomes, including mortality, recurrent thromboembolic events, and treatment-related complications, were assessed throughout this period.

 Upon discharge, the majority of patients (54.7%) were prescribed warfarin, while 43.2% received a novel oral anticoagulant (NOAC). A small proportion (2.1%) were treated with enoxaparin. Anticoagulant therapy was maintained for a mean duration of 8.7 months, with a minimum treatment period of two months.

 Echocardiography was performed. The mean ejection fraction was 52.5% (SD: ± 6.1%). Right ventricular enlargement was prevalent, observed in 55.8% of the cohort. Furthermore, impaired right ventricular function was noted in 49% of the patients. Mean systolic pulmonary artery pressure during the follow-up period was 22.1 mm Hg (SD: ± 9.8), with a maximum recorded value of 38 mm Hg.

 Significant clinical outcomes included the development of chronic thromboembolic pulmonary hypertension (CTEPH) in two patients and the onset of heart failure in four patients.

###  Analysis of Mortality Risk Factors 

 This study investigated the relationship between various clinical variables and mortality in patients diagnosed with PE. Analysis of in-hospital mortality revealed that while 55% of the 11 patients who died were over 65 years of age, this was not statistically significant (*P* = 0.492). Conversely, advanced age was significantly associated with mortality during the follow-up period, with 78% of deaths occurring in patients over 65 (*P* = 0.002).

 A statistically significant majority of in-hospital deaths were female (82%, *P* = 0.029), whereas sex was not a significant predictor of mortality in the follow-up period (*P* = 0.28). All in-hospital deaths occurred in patients with provoked PE (*P* = 0.006), and 82% were attributed to massive PE (*P* = 0.014). Absence of thrombolytic therapy was significantly correlated with in-hospital mortality (*P* = 0.001) but did not significantly affect mortality during follow-up (*P* = 0.65). Furthermore, 73% of patients who died in-hospital exhibited a left ventricular ejection fraction (LVEF) below 50% (*P* < 0.001). The presence of comorbidities was significantly associated with mortality in the follow-up period (*P* = 0.029) (Table 2).

**Table 2:** Association of Clinical Variables with Mortality Among Patients with Pulmonary Embolism

**Variable**	**In-hospital Death (n=11)**	***P***	**Follow-up Death (n=18)**	***P***
	**No. (%)**		**No. (%)**	
Age > 65 years				
Yes	6 (55)	0.492	14 (78)	0.002
No	5 (45)		4 (22)	
Sex				
Male	2 (18)	0.029	8 (44)	0.28
Female	9 (82)		10 (56)	
Provoked				
Yes	11 (100)	0.006	13 (72)	0.21
No	0 (0)		5 (28)	
Massive PE				
Yes	9 (82)	0.014	6 (33)	0.86
No	2 (18)		13 (67)	
Thrombolytic therapy				
Yes	8 (73)	0.001	12 (67)	0.65
No	3 (27)		6 (33)	
LVEF ≥ 50%				
Yes	3 (27)	0	14 (82)	0.37
No	8 (73)		3 (18)	
PASP < 40				
Yes	3 (29)	0.77	12 (67)	0.33
No	8 (71)		6 (33)	
Comorbidity^*^				
Yes	8 (73)	0.298	34 (56)	0.029
No	3 (27)		27 (44)	

PE: pulmonary embolism; LVEF: left ventricular ejection fraction; PASP: pulmonary arterial systolic pressure. “Yes” and “No” indicate the presence or absence of a particular characteristic or risk factor. ^*^Comorbidities (HTN, DM, IHD, HLP, cancer, CVA, and lung disease).

###  Predictive Factors for Mortality 

 A hierarchical logistic regression model was utilized to ascertain the factors predictive of in-hospital and follow-up mortality in a cohort of patients diagnosed with PE. Of the 109 patients initially identified, 103 (94.5%) were included in the final analysis. Six cases (5.5%) were excluded due to incomplete data.

 The model was constructed in a stepwise fashion, incorporating the following variables sequentially: age, sex, and PE severity. Model fit improved significantly with each step, as evidenced by the reduction in -2 Log likelihood values (from 106.322 to 91.108) and the concomitant increase in Nagelkerke R^2^ (from 0.138 to 0.321). The final model accounted for approximately 32.1% of the variance observed in mortality.

 In the final model, age emerged as a significant predictor of mortality (B = 0.063, *P* < 0.001), with each additional year of age associated with a 6.5% increase in the odds of death (odds ratio [OR] = 1.065). Female patients exhibited significantly higher odds of mortality compared to their male counterparts (B = 1.486, *P* = 0.009; OR = 4.421). Conversely, lower PE severity was associated with a reduction in mortality odds (B = -1.338, *P* = 0.023; OR = 0.262). Other variables, including etiology of PE, administration of thrombolytic therapy, and comorbidity burden and duration of oral anticoagulant therapy did not achieve statistical significance and failed to enhance the predictive power of the model (Table 3).

**Table 3:** Independent Predictors of Mortality in Patients with Pulmonary Embolism

**Predictor**	**B**	**SE**	**OR**	**95% CI**	***P*** ** value**
Sex (Female)	1.486	0.567	4.4	1.4-13.4	0.009
Non-massive PE	-1.338	0.590	0.26	0.08-0.8	0.023
Age > 65 years	0.063	0.017	1.06	1.0-1.1	0.000
Constant	-6.572	1.623	0.001	1.4-13.4	0.000

B: Regression coefficient, SE: Standard error, OR: Odds ratio, CI: Confidence interval, PE: pulmonary embolism.

###  Survival Analysis

 Kaplan-Meier survival analysis was employed to investigate the influence of various factors on mortality rates in a cohort of patients diagnosed with PE. The analysis revealed several significant associations with survival outcomes.

**Figure 1:**
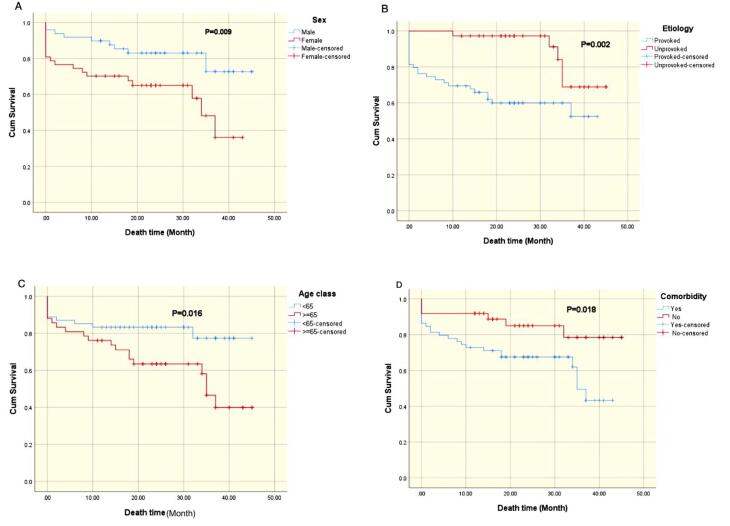
Kaplan-Meier Survival Curves of Mortality after Pulmonary Embolism. (A) Sex, (B) Etiology, (C) Age, and (D) Comorbidity

 Sex: Male sex emerged as a significant predictor of improved survival compared to female sex (*P* = 0.009), as illustrated in Figure 1A.

 Etiology of PE: The underlying cause of PE also significantly affected survival (*P* = 0.002). Patients with unprovoked PE exhibited a survival advantage compared to those with provoked PE (Figure 1B).

 Age: Age was identified as a significant prognostic factor (*P* = 0.016). Patients younger than 65 years demonstrated superior survival rates relative to their older counterparts (Figure 1C).

 Comorbidities: The presence of comorbidities was significantly associated with reduced survival (p = 0.018), as depicted in Figure 1D.

 Data Completeness: Complete follow-up data were available for 96 patients.

###  Bleeding Complications

 A total of 11 patients (10.1%) experienced bleeding complications during the study. During the hospitalization period, 10 out of 109 patients (9.1%) presented with bleeding. In the follow-up period, this decreased to 1 out of 96 patients (1.0%) (Table 4).

**Table 4:** Complications During hospitalization and follow-up for patients with Pulmonary Embolism

**Complication**	**In hospital ** **No. (%)**	**Follow up** **No. (%)**	**Total** **No. (%)**
Bleeding	10 (9.1)	1 (1.0)	11(10.1)
Recurrence of PE	-	1 (1.0)	1 (1.0)
CTEPH	-	2 (2.1)	2 (2.1)
Death	11 (10.1)	18 (18.4)	29 (26.6)
Total	21 (19.2)	22 (22.4)	43

CTEPH: chronic thromboembolic pulmonary hypertension, PE: pulmonary embolism.

 The observed bleeding complications included gastrointestinal bleeding, hematoma formation at the intravenous line insertion site, vaginal bleeding, and a single instance of intracerebral hemorrhage. Notably, none of the bleeding events resulted in mortality. The most frequently observed complication was hematoma formation, which occurred in 4.6% of the patient population.

## Discussion

 This study provides valuable insights into the clinical presentation and outcomes of patients diagnosed with PE. Analysis of baseline characteristics revealed significant trends. The mean age of the patient cohort was 59 years, with a male predominance. The most frequently reported symptom was dyspnea, followed by chest pain. These findings align with the existing literature, which identifies these symptoms as hallmark indicators of PE.^7-9^ Furthermore, a substantial proportion of patients exhibited electrocardiogram (ECG) abnormalities, underscoring the significant cardiac involvement associated with this condition.

 This study investigated the diagnostic modalities employed in the assessment of PE. Pulmonary computed tomographic angiography (CTPA) was the most frequently utilized diagnostic tool, followed by clinical evaluation and ventilation-perfusion (V/Q) scans. This diagnostic pattern is consistent with established clinical guidelines, which advocate for CTPA as the preferred imaging modality for PE diagnosis.^10^ The analysis of PE subtypes revealed a predominance of sub-massive PE, indicating a substantial burden of disease severity within the study cohort.

 We observed a noteworthy in-hospital mortality rate of 10%, with eleven patient deaths occurring despite diagnosis and treatment. Statistical analysis revealed several significant predictors associated with in-hospital mortality, including provoked PE, massive PE, absence of thrombolytic therapy, and LVEF below 50%. These findings emphasize the critical importance of prompt and appropriate therapeutic intervention in PE cases and further highlight the potential impact of underlying cardiac dysfunction on patient prognosis.^11,12^

 In this study, the observed mortality rate increased to 18.4% during the follow-up period, with 18 patient deaths recorded. Advanced age emerged as a significant predictor of mid-term mortality (*P* < 0.05), underscoring the impact of age-related physiological factors on long-term patient outcomes. Notably, the etiology of PE (provoked versus unprovoked) did not significantly influence mid-term mortality, suggesting that alternative factors may be contributing to mortality risk in the post-hospitalization period. Furthermore, neither the administration of thrombolytic therapy nor baseline measures of LVEF and systolic pulmonary artery pressure demonstrated significant associations with mid-term mortality. This observation indicates that the prognostic value of these factors may differ between short-term and long-term outcomes, warranting further investigation.^13^

 A subset of patients in this study experienced bleeding complications both during hospitalization and follow-up. The cumulative incidence of bleeding complications was 10.1%, with a single case reported during the follow-up period. Observed bleeding events included gastrointestinal bleeding, hematoma formation at the intravenous line insertion site, vaginal bleeding, and intracerebral hemorrhage. Notably, none of the reported bleeding events resulted in mortality. These findings underscore the importance of vigilant monitoring and meticulous management of anticoagulation therapy to minimize the risk of bleeding in patients receiving treatment for PE.^14,15^

 This study offers valuable insights into the management of patients with PE. The patients were discharged on anticoagulation therapy, with either warfarin or a non-vitamin K antagonist oral anticoagulant (NOAC) prescribed. The mean duration of anticoagulation (8.7 months) aligned with established clinical guidelines for PE management.^16-18^ The echocardiographic assessment revealed right ventricular dysfunction in nearly half of the patient cohort, underscoring the importance of cardiac function monitoring during follow-up.^19,20^. Furthermore, two patients developed CTEPH, highlighting the potential for long-term complications and the need for ongoing surveillance in this patient population.^21,22^

 We observed a persistently elevated mortality risk in patients following acute PE throughout the entire follow-up duration. This sustained risk signifies a protracted threat to long-term survival post-PE. Factors such as the initial severity of the embolic event and the presence of comorbidities, particularly cardiovascular disease, are associated with this increased mortality risk. These findings are consistent with those of Ng et al,^23^ who, in a study of 1023 patients with a mean follow-up of 3.8 years, reported a 35.5% mortality rate. Notably, post-discharge mortality in this cohort was 2.5 times higher than that observed in an age- and sex-matched general population. These results underscore the critical need for implementation of long-term surveillance strategies to mitigate the substantial mortality risk faced by patients in the post-PE period.

 This study is subject to several limitations. Firstly, the sample size of 109 patients was relatively small, potentially limiting the generalizability of the findings to larger populations. Secondly, the retrospective, single-center design introduces inherent biases and restricts the capacity to draw definitive causal inferences. Absence of a control group further complicates the assessment of causality between observed variables and mortality outcomes. Thirdly, the short follow-up period may not have captured the full spectrum of long-term outcomes associated with PE. Additionally, the study did not account for potentially significant variables such as socioeconomic status and N-terminal prohormone of brain natriuretic peptide (NT-ProBNP) levels, which could influence patient outcomes. The influence of confounding factors, including age and gender, may have obscured the intricate relationships between specific PE characteristics and mortality. Future research with larger, prospective, multicenter designs incorporating a wider range of variables is warranted to address these limitations and provide more robust conclusions.

## Conclusion

 This study contributes valuable insights into the clinical presentation, mortality rates, and potential risk factors associated with PE. The results underscore the critical importance of prompt diagnosis, appropriate therapeutic intervention, and vigilant management of patients presenting with this condition to optimize outcomes and mitigate mortality risk. Further investigation, particularly through large-scale studies, is warranted to validate these findings and delve into additional factors that may influence mortality outcomes in individuals diagnosed with PE.
